# Transcutaneous immunotherapy via laser-generated micropores efficiently alleviates allergic asthma in Phl p 5–sensitized mice

**DOI:** 10.1111/all.12005

**Published:** 2012-09-05

**Authors:** D Bach, R Weiss, M Hessenberger, S Kitzmueller, E E Weinberger, W D Krautgartner, C Hauser-Kronberger, C Boehler, J Thalhamer, S Scheiblhofer

**Affiliations:** 1 Division of Allergy and Immunology, Department of Molecular Biology, University of SalzburgSalzburg, Austria; 2 Division of Light & Electron Microscopy, Department of Organismic Biology, University of SalzburgSalzburg, Austria; 3 Department of Pathology, University Hospital Salzburg, Paracelsus Medical UniversitySalzburg, Austria; 4 Pantec Biosolutions AGRuggell, Liechtenstein

**Keywords:** allergy, immunotherapy, laser, micropores, transcutaneous

## Abstract

**Background:**

Specific immunotherapy via the subcutaneous or oral route is associated with local and, in some cases, systemic side effects and suffers from low patient compliance. Due to its unique immunological features, the skin represents a promising target tissue for effective and painless treatment of type I allergy. The current study was performed to compare the efficacy of transcutaneous immunotherapy via laser-generated micropores to subcutaneous injection.

**Methods:**

BALB/c mice were sensitized by intraperitoneal injection of recombinant grass pollen allergen Phl p 5 together with alum. Subsequently, lung inflammation was induced by repeated intranasal challenge. During the treatment phase, adjuvant-free Phl p 5 was applied in solution to microporated skin or was subcutaneously injected. Lung function and cellular infiltration; Phl p 5–specific serum levels of IgG1, IgG2a, and IgE; and cytokine levels in bronchoalveolar lavage fluids as well as in supernatants of splenocyte cultures were assessed.

**Results:**

Both therapeutic approaches reduced airway hyperresponsiveness and leukocyte infiltration into the lungs. Whereas subcutaneous immunotherapy induced a systemic increase in Th2-associated cytokine secretion, transcutaneous application revealed a general downregulation of Th1/Th2/Th17 responses. Successful therapy was associated with induction of IgG2a and an increase in FOXP3+ CD4+ T cells.

**Conclusions:**

Transcutaneous immunotherapy via laser microporation is equally efficient compared with conventional subcutaneous treatment but avoids therapy-associated boosting of systemic Th2 immunity. Immunotherapy via laser-microporated skin combines a painless application route with the high efficacy known from subcutaneous injections and therefore represents a promising alternative to established forms of immunotherapy.

With a prevalence of up to 30% in developed countries, IgE-mediated allergic diseases have become a major burden for public health systems 1. Introduced 100 years ago 2–4, specific immunotherapy (SIT) still is the only causal treatment for patients suffering from rhinoconjunctivitis, asthma, or hypersensitivity to insect venom, by redirecting inappropriate T helper 2 (Th2)-driven immune responses against allergens. Despite its proven clinical efficacy 5,6, only a small percentage of allergic patients decide to undergo SIT instead of symptomatic treatment 7,8. In clinical practice, SIT is mostly performed by 50–80 subcutaneous injections (SCIT) of gradually increasing allergen doses over 3–5 years, leading to poor compliance rates 9. Also, the acceptance of SCIT is limited by local or systemic allergic side effects 10. As a needle-free alternative, sublingual immunotherapy (SLIT) with drops or tablets has been approved 11; however, SLIT requires daily intake of large amounts of allergen with considerable costs, offers no reduced treatment duration, and is frequently accompanied by oral as well as gastrointestinal side effects 12. Despite the opportunity for self-administration, SLIT has low patient compliance rates 13. Furthermore, SLIT might be less effective due to poor allergen uptake caused by short contact with the oral mucosa 14.

First described by Streilein et al. as an organ with important immunological functions 1983, the skin represents an attractive target tissue for vaccine delivery. It is rich in immunocompetent cells, including Langerhans cells, dermal dendritic cells, and keratinocytes, and is efficiently drained by the lymphatic system 16–18. Epicutaneous immunotherapy (EPIT), already employed in the 1950s 19,20, was recently revisited in animal models and clinical studies by the application of allergen extracts via adhesive patches to either untreated or tape-stripped skin 21–26.

In the present study, we explore for the first time the potential of transcutaneous immunotherapy via laser-generated micropores. Employing the P.L.E.A.S.E.® (Precise Laser Epidermal System; Pantec Biosolutions AG, Ruggell, Liechtenstein) infrared laser device, aqueous micropores of variable number, density, and depth can be created at a defined skin area 27,28, followed by the application of allergen in solution. Compared to SCIT, this approach proved to be at least equally therapeutically effective in a mouse model of grass pollen allergy, while avoiding a therapy-induced boost of Th2 cytokines. Our findings establish laser microporation as a novel delivery platform for transcutaneous immunotherapy (TCIT).

## Methods

### Mice and treatments

BALB/c females, aged 6–8 weeks, were purchased from Charles River Laboratories (Sulzfeld im Grabfeld, Germany). Ovalbumin (OVA) transgenic DO11.10 and BALB/c mice expressing the CD45.1 alloantigen were bred and maintained at the animal facility of the University of Salzburg and used at an age of 6–12 weeks. DO11.10 mice were crossed with CD45.1 BALB/c, and the F1 generation served as donors for lymphocyte transfers. All animal experiments were conducted according to local guidelines approved by the Austrian Ministry of Science (Permit Number: GZ 66.012/0004-II/10b/2010). Mice were sensitized on days 0 and 10 by intraperitoneal injections with 1 μg recombinant Phl p 5 (Biomay AG, Vienna, Austria) in 100 μl PBS adjuvanted with 100 μl Al(OH)3, followed by intranasal instillations of 1 μg Phl p 5 in 40 μl PBS divided between both nares on days 31, 32, 33, 38, 39, and 40. Specific immunotherapy was performed twice a week for three weeks (days 55, 59, 62, 66, 69, and 73) by application of 50 μg recombinant Phl p 5 in 80 μl PBS to microporated skin areas or subcutaneous (s.c.) injection of the same amount. For challenge, another two series of intranasal instillations were performed on days 80, 81, 82, and 87, 88, 89, respectively, and the animals were killed after invasive measurement of lung functions on day 90. Blood samples were collected on days 41 (after sensitization), 80 (after therapy), and 90 (after intranasal challenge). An overview on the experimental schedule is given in Fig. 3A.

### Laser microporation

The day before laser microporation, animals were shaved on their back with a clipper, and depilatory cream was used to remove residual hair. The P.L.E.A.S.E.® device (Pantec Biosolutions AG) used for microporation contains a diode-pumped Er:YAG laser that emits light at 2.94 μm, corresponding to a major absorption peak of water molecules present in the skin. Their excitation and explosive evaporation lead to fractional ablation of the skin and the formation of micropores with a diameter of approximately 150 μm. Due to the high-energy, short-duration laser pulses, heat transfer to neighboring tissue is negligible. The P.L.E.A.S.E.® system employs a scanning laser technique to create an array of micropores with user-defined number and depth 29.

Microporation was performed by placing anesthetized mice with their back at the focal length of the laser. Laser parameters, that is, number of pores/cm^2^, number of pulses per pore, and fluence (energy applied per unit area) were preprogrammed using the device software. For transcutaneous immunotherapy, four pulses with a fluence of 1.9 J/cm^2^ per pulse were applied, and 500 pores/cm^2^ (circular area, 1 cm diameter) were generated. Phl p 5 or OVA (grade V; Sigma-Aldrich, Deisenhofen, Germany) was applied as aqueous solution to the microporated skin areas, where it was completely absorbed within 5–10 min.

### Histological analysis

The 2-μm paraffin sections of skin samples were prepared and stained with hematoxylin/eosin using standard methods.

For scanning electron microscopy, samples were fixed for 2 h with Karnovsky 30, and postfixation was performed with 1% osmium tetroxide (buffered at pH 6.5 with 0.1 M sodium cacodylate) for further 2 h. The postfixed samples were dehydrated in an ascending series of ethyl alcohol, critical-point-dried, and subsequently sputtered with gold (~5 nm) and analyzed in an environmental scanning electron microscope, ESEM XL30 (FEI; Philips, Eindhoven, the Netherlands), operating at 20 kV.

### *In vivo* proliferation of OVA-transgenic T cells

For *in vivo* proliferation assay, on day 0, 2 × 10^6^ carboxyfluorescein diacetate succinimidyl ester (CFSE)-labeled splenocytes from DO.11.10 donors (CD45.1 background) were adoptively transferred to naive recipient mice as described 31. 20 μg of OVA (2 mg/ml in PBS) was applied to laser-microporated skin (900 pores, 1.5 cm diameter, six pulses delivered at 1.9 J/cm^2^ per pulse) on day 1. Six days later, draining lymph node cells were prepared, recorded on a FACSCanto II flow cytometer, and analyzed using FACSDiva Software (BD Biosciences, Schwechat, Austria). Proliferation was assessed by gating on CD45.1+ CD4+ cells and calculating the proliferation index (proliferated/nonproliferated cells).

### Serology, cytokines, and flow cytometry

Sera were analyzed for Phl p 5–specific IgG, IgG1, and IgG2a by a luminescence-based ELISA at indicated serum dilutions lying within the linear range of the assay. Biologically functional IgE was determined by an *in vitro* basophil release assay as described 32. Splenocytes were cultured in the presence of 10 μg/ml recombinant Phl p 5 or a mixture of six immunodominant Phl p 5 peptides 33 (P154-168 [ATLSEALRIIAGTLE], P196-210 [AFKVAATAANAAPAN], P214-228 [TVFEAAFNDAIKAST], P232-246 [YESYKFIPALEAAVK], P250-264 [AATVATAPEVKYTVF], P268-282 [LKKAITAMSEAQKAA]) each applied at 1 μM for 3 days, and cytokine profiles in supernatants thereof were assessed via mouse Th1/Th2/Th17/Th22 13plex FlowCytomix multiplex kit combined with the mouse GM-CSF FlowCytomix simplex kit (eBioscience, San Diego, CA, USA) according to the manufacturer's instructions. Additionally, TGF-β1 was measured using a human/mouse TGF-β1 ELISA Ready-SET-Go! kit (eBioscience).

Re-stimulated splenocytes were harvested, washed once with FACS buffer (PBS, 1% BSA, 2 mM EDTA), and stained for 10 min on ice with anti-CD4-FITC (eBioscience). After washing, FOXP3 was stained using the FOXP3 Fixation/Permeabilization Concentrate and Diluent, and Anti-Mouse/Rat FOXP3 PerCP-Cy5.5 (both eBioscience) according to the manufacturer's protocol.

Cells were then resuspended in FACS buffer and analyzed on a FACS Canto II flow cytometer using FACS diva software. Live lymphocytes were gated on FSC/SSC plots, and the percentage of FOXP3+ CD4+ T cells was assessed.

### Lung parameters

Airway hyperreactivity (AHR) was assessed via measurement of Penh by unrestrained whole-body plethysmography (Buxco, Winchester, UK) before and after specific immunotherapy 34. At the end of the experimental schedule, invasive measurement of pulmonary resistance and dynamic compliance was performed with a FinePointe™ RC system for mice (Buxco). Animals were challenged with increasing doses of methacholine in 0.9% NaCl, and flow and pressure signals were analyzed using biosystem XA software (Buxco) 34. Subsequently, bronchoalveolar lavages were performed as described 35. Cells were stained with CD45-FITC, CD4-APC/Cy7, CD19-PE, and Gr1-APC (Biolegend or eBioscience, San Diego, CA, USA). Eosinophils were distinguished from other leukocyte populations by their CD45^med^ Gr1^low^ side-scatter^high^ phenotype. Cytokine profiles of lavage fluids were established using the mouse Th1/Th2/Th17/Th22 13plex FlowCytomix multiplex kit (eBioscience).

### Statistical analysis

Data presented for therapy are derived from two independent experiments with six mice per group with similar results. Pooled data from both experiments (*n* = 12) were analyzed and are shown as individual data points or means ± SEM.Statistical significance between groups was assessed by one-way anova followed by Newmann–Keuls *post hoc* test (alpha = 0.05) using GraphPad Prism 5.

## Results

### Laser microporation induces immune responses in a pore depth-dependent manner

**Figure 1:**
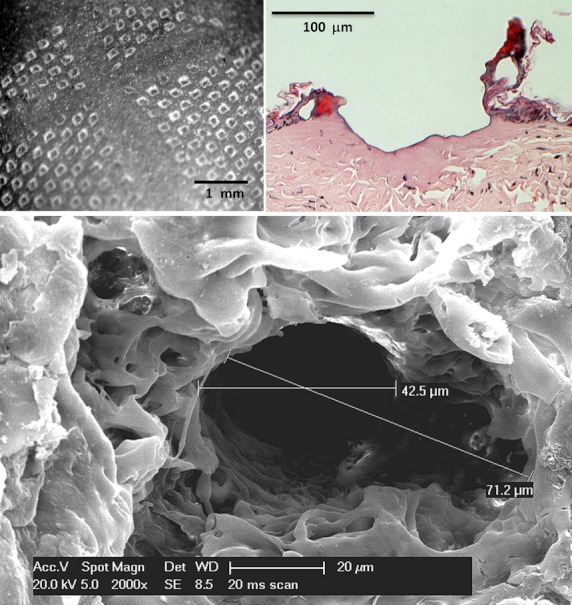
Histological analysis of laser-microporated mouse skin. Upper left: Top view of an array of micropores (500 pores/cm^2^). Paraffin section and scanning electron microscopy picture of a single micropore generated with four laser pulses delivered at 1.9 J/cm^2^/pulse (upper right) or eight laser pulses delivered at 0.76 J/cm^2^ per pulse (bottom).

**Figure 2:**
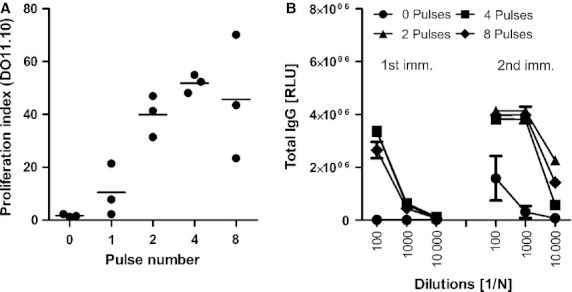
Immune responses after transcutaneous immunization via laser-microporated skin. Proliferation of adoptively transferred OVA-transgenic DO11.10 cells after OVA application to the micropore arrays (A). Phl p 5–specific IgG responses after one or two applications of recombinant Phl p 5 to skin microporated using various pulse numbers (B). Data are shown as proliferation indices or relative light units (*n* = 3, means ± SEM). Panel A reproduced from 36.

Controlled dermal ablation using the P.L.E.A.S.E. device generated an array of micropores of precise depth and position. Scanning electron microscopy shows the accessibility of individual cell layers, allowing for high diffusion rates, no thermal damage of the tissue was observed (Fig. 1), and full re-epithelialization was achieved within 2 days 36. Soluble antigen (OVA) applied to microporated skin was quickly absorbed and induced proliferation of adoptively transferred OVA-specific DO11.10 cells in a pore depth-dependent manner, with a maximal proliferation induced by 4–6 pulses (Fig. 2A). Higher pulse numbers led to inconsistent results, probably due to beginning tissue carbonization, and thereby reduced antigen uptake 36. Additionally, application of recombinant Phl p 5 to microporated, but not intact skin, induced antibody induction after a single immunization and significantly higher antibody titers after a booster immunization (*P* < 0.01; Fig. 2B).

### TCIT and SCIT boost IgG but do not affect IgE levels

**Figure 3:**
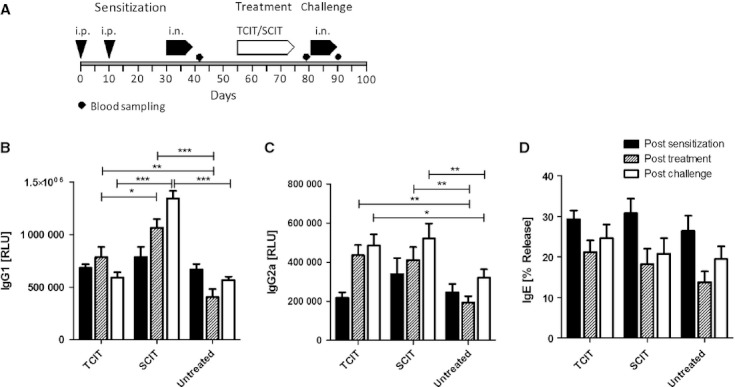
Serological changes after immunotherapy of Phl p 5 sensitized mice. (A) Schematic overview of experimental schedule. Phl p 5–specific IgG1 (B), IgG2a (C), and IgE (D) after sensitization (black bars), after therapeutic intervention (hatched bars), and after intranasal re-challenge (open bars). Data are shown as means ± SEM (*n* = 12). TCIT: transcutaneous immunotherapy; SCIT: subcutaneous immunotherapy. *P < 0.05, **P < 0.01, ***P < 0.001.

We investigated transcutaneous immunotherapy via microporated skin (TCIT) in a mouse model of allergic asthma and compared its outcomes with classical subcutaneous injections (SCIT) using the same amount of antigen (50 μg). Mice were sensitized by intraperitoneal injection of Phl p 5 absorbed to alum, and lung inflammation was induced by repeated intranasal instillation of allergen. After six treatments over a period of 3 weeks, mice were intranasally re-challenged. See Fig. 3A for the experimental schedule. Mice displayed similar levels of Phl p 5–specific IgG1, IgG2a, and IgE postsensitization. Treatment induced a significant boost of IgG2a (Fig. 3C) compared with the untreated control group, with a more pronounced increase in the TCIT group. The boost of IgG1 was restricted to the SCIT group (Fig. 3B). IgE was neither increased by SCIT nor by TCIT, but reduced in all groups (including the untreated control group), as measured by a basophil release assay (Fig. 3D).

### TCIT and SCIT alleviate airway hyperresponsiveness and leukocyte infiltration into the lung

**Figure 4:**
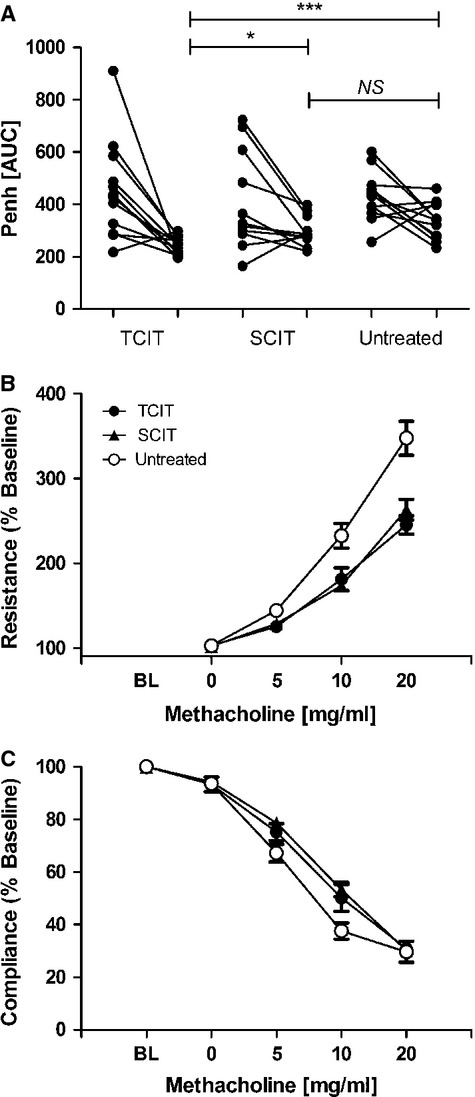
Airway hyperresponsiveness (AHR) after immunotherapy of Phl p 5–sensitized mice. AHR was assessed before and after transcutaneous (TCIT) or subcutaneous (SCIT) immunotherapy via whole-body plethysmography (A) or by invasive measurement of lung resistance (B) and dynamic compliance (C). Data are shown as individual data points or means ± SEM (*n* = 12). BL = baseline measurement; **P < 0.01, ***P < 0.001.

**Figure 5:**
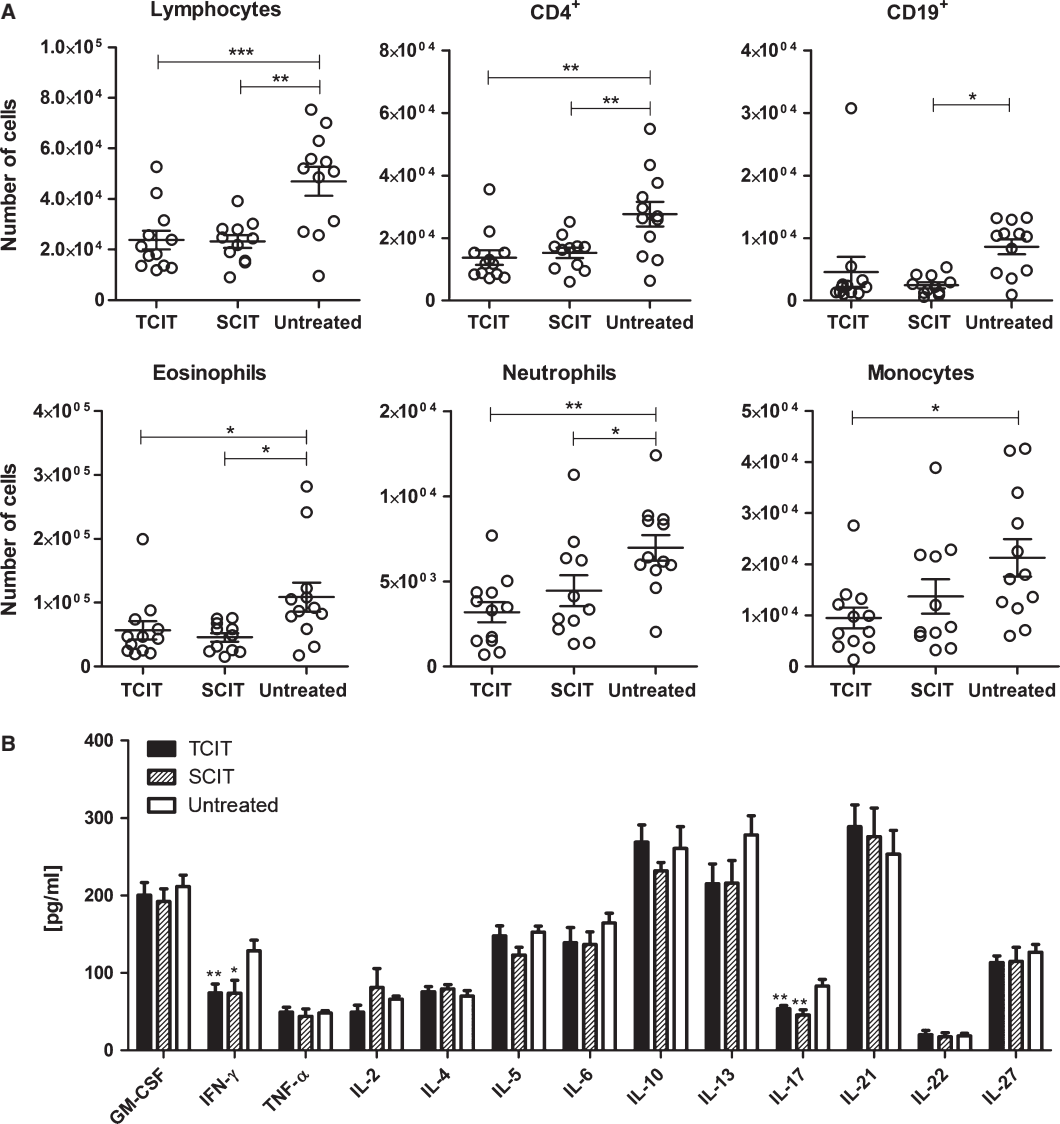
Cellular composition and cytokine levels in BALF after immunotherapy of Phl p 5–sensitized mice. Total number of different leukocyte populations per BAL (A) and cytokine levels (B) are shown as individual data points and/or means ± SEM (*n* = 12) of mice treated by transcutaneous (TCIT) or subcutaneous (SCIT) immunotherapy. *P < 0.05, **P < 0.01, ***P < 0.001.

Airway hyperresponsiveness (AHR) was indirectly assessed via unrestrained whole-body plethysmography after sensitization and after therapy (postchallenge). As shown in Fig. 4A, TCIT induced a significant reduction of Penh compared to SCIT (*P* < 0.05) and untreated controls (*P* < 0.001). Penh results were confirmed by invasive measurement of lung resistance and dynamic compliance. Here, TCIT and SCIT performed equally well, both significantly reducing lung resistance (*R*) (Fig. 4B) while increasing dynamic compliance (*C*_dyn_) (Fig. 4C). These results correlated with a reduction of all measured leukocyte populations in bronchoalveolar lavage fluid (BALF), including T helper cells, B cells, eosinophils, neutrophils, and monocytes (Fig. 5A). Despite this notable reduction in BAL leukocytes, cytokine levels in BALF were only mildly affected. Of 13 detected cytokines, only IFN-γ and IL-17 were significantly suppressed after TCIT as well as SCIT (Fig. 5B); however, we also observed a tendency toward decreased levels of BAL IL-13, which has been described as a major factor for leukocyte infiltration in a secondary allergen challenge 37. Analysis of HE-stained paraffin sections revealed a nonsignificant reduction of peribronchial and perivascular infiltration of mononuclear cells compared with untreated control animals for both treatments (Fig. S1).

### TCIT suppresses inflammatory cytokines while SCIT boosts Th2 cytokines

**Figure 6:**
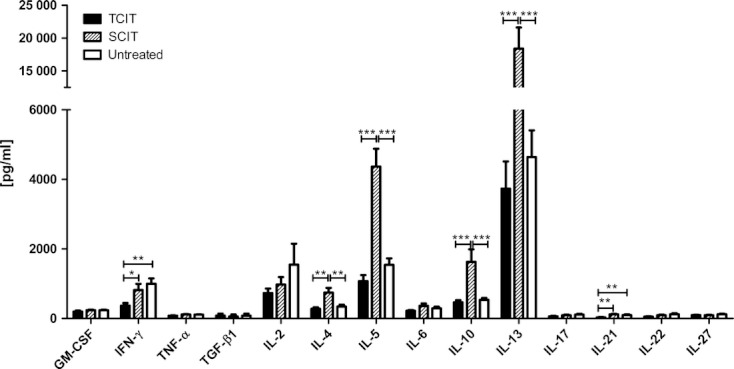
Cytokine levels in supernatants of re-stimulated splenocytes taken after transcutaneous (TCIT) or subcutaneous (SCIT) immunotherapy of Phl p 5–sensitized mice. Data are shown as means ± SEM (*n* = 12). *P < 0.05, **P < 0.01, ***P < 0.001.

**Figure 7:**
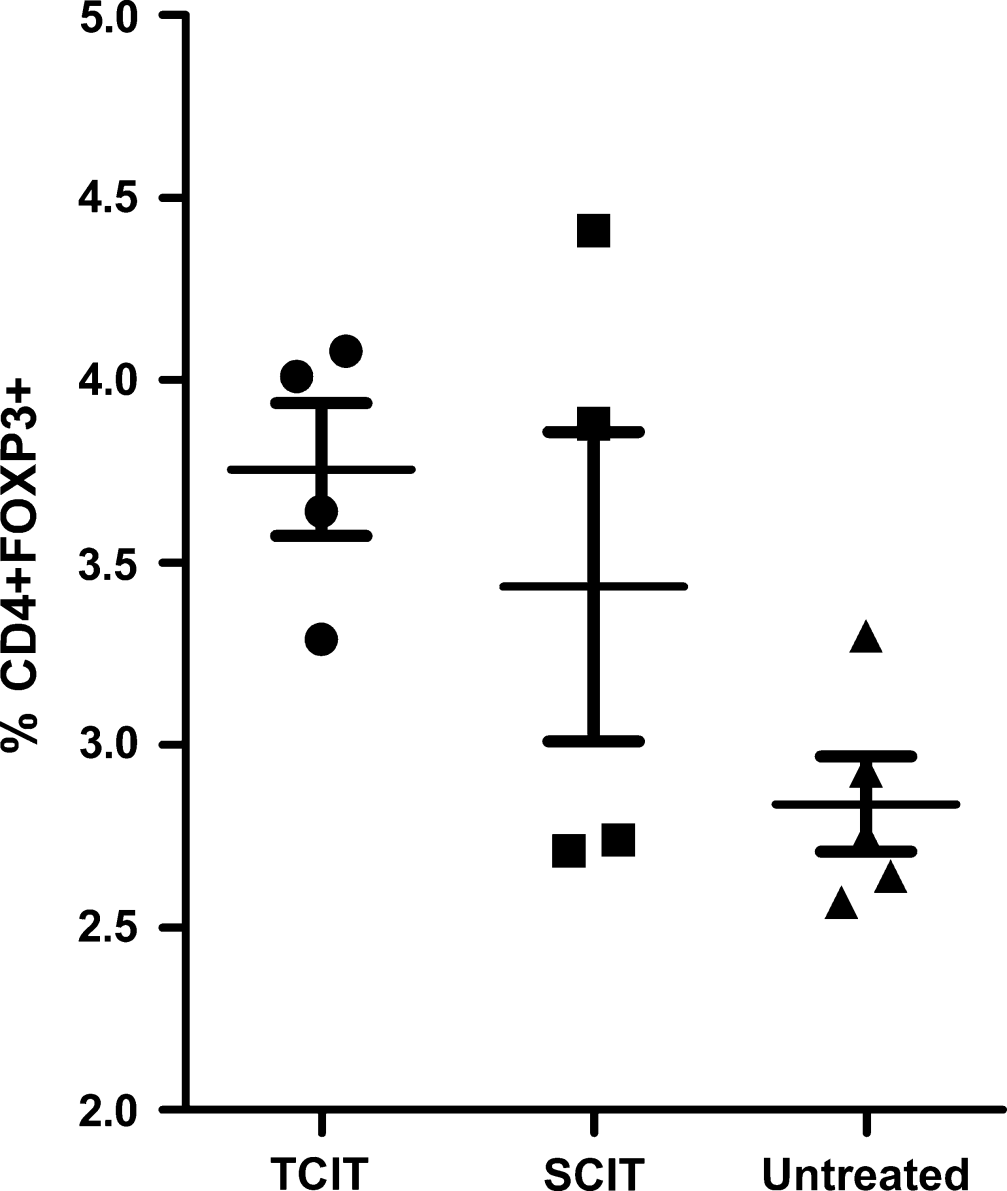
Percentage of FOXP3+ CD4+ T cells of total lymphocytes after re-stimulation with recombinant Phl p 5. TCIT = transcutaneous immunotherapy; SCIT = subcutaneous immunotherapy.

In order to study the systemic immune status after TCIT or SCIT followed by a final challenge with allergen, cytokine secretion by *in vitro* re-stimulation of splenocytes with a cocktail of peptides reflecting the major murine CD4 T cell epitopes was assessed. We found that TCIT significantly suppressed the pro-inflammatory cytokines IFN-γ and IL-21 compared with untreated as well as SCIT animals. In contrast, SCIT, but not TCIT, resulted in a striking boost of the Th2 cytokines IL-4, IL-5, IL-10, and IL-13 (Fig. 6). Suppression of cytokine responses after TCIT was associated with an increase in the percentage of FOXP3+ CD4+ T cells compared with untreated mice (Fig. 7).

## Discussion

Increasing knowledge about skin immunology has recently fueled efforts to develop novel strategies for transcutaneous vaccination 38. The ideal target tissue for specific immunotherapy of type I allergies has to be easily accessible, rich in immunocompetent cells, and nonvascularized to avoid direct contact of allergen with the general circulation. The skin harbors high numbers of professional antigen-presenting cells, such as Langerhans cells of the epidermis and dermal dendritic cells for antigen uptake and presentation 17, and keratinocytes, which potentiate and control immune responses by cytokine and chemokine production 16. It has been demonstrated that skin vaccination results in highly efficient antigen trafficking into lymph nodes 18, whereas after subcutaneous immunization, <1% of proteins end up in the draining lymph nodes 39. The major challenge for cutaneous immunization is to penetrate the top layer of the skin, the stratum corneum, which acts as a barrier for diffusion. Furthermore, cutaneous immunization must be highly efficient in order to reduce the duration of therapy and should employ a patient-friendly, needle-free, and painless technique.

Epicutaneous immunotherapy has been recently investigated in mouse models and clinical studies 21–25. In mice, application of antigen to slightly barrier-disrupted skin, and even intact skin, can be sufficient to induce therapeutic effects. However, it is questionable, whether these approaches would result in adequate and reproducible effects in humans, who have a much thicker and less hairy skin. Moreover, the human skin is an elastic, heterogeneous tissue, and its mechanical and structural properties vary considerably with age, hydration level, body location, skin type, body weight, and ethnicity 40,41. These facts may provide an explanation for the still improvable therapeutic effects in previous clinical studies of EPIT 21,22,24 which required high allergen doses 25. However, application of high doses of allergen to large areas of barrier-disrupted skin for prolonged periods of time may trigger local adverse effects 24.

In contrast, the P.L.E.A.S.E.® device, which we used in the present study, provides the opportunity to circumvent the stratum corneum in a highly adjustable manner, even allowing for selective targeting of different skin layers 36. It has been demonstrated that this delivery platform significantly enhances transport rates of low weight molecular compounds 27,28,42 and also therapeutic antibodies through human and porcine skin samples 29. Recently, delivery of follicle stimulating hormone via laser-generated micropores has been shown in a clinical study 43.

Our current work demonstrates that laser poration can generate adjustable arrays of precise micropores of defined depth (Fig. 1). At 500 pores/cm^2^, only 7% of the porated area is open for diffusion, whereas the remainder of the skin is intact. As shown in Fig. 2A, proliferation of adoptively transferred OVA-transgenic DO11.10 cells increases with the number of laser pulses, indicating enhanced antigen uptake that finally reaches a plateau and can even decline due to carbonization of pores (data not shown). Interestingly, the results in Fig. 2B demonstrate that two applications of antigen on solely depilated mouse skin are sufficient to trigger a detectable antibody response. This can be explained by the very thin stratum corneum/epidermis of mice (compared with humans). Nevertheless, also in mice, laser microporation is necessary for induction of strong cellular and humoral immune responses (Fig. 2).

With respect to functional lung parameters, TCIT showed similar (Fig. 4B,C, and Fig. 5) or even superior (Fig. 4A) therapeutic efficacy compared with SCIT and, in contrast to SCIT, did not result in a systemic boost of Th2-associated cytokines (Fig. 6). It has been suggested that barrier disruption of the skin by tape stripping activates keratinocytes and Langerhans cells, resulting in predominant Th2/Treg responses, whereas penetration into deeper skin layers (‘deep epithelial trauma’) imprints tissue-resident DCs to induce Th1 responses 44. In our experiments, we used a pore depth of approximately 30–40 μm, thus bypassing the epidermis and delivering the antigen directly into the dermal compartment. Although we measured a downregulation of Th1/Th17-associated cytokines and unaltered levels of Th2 cytokines after TCIT (Fig. 6), we also detected induction of IgG2a, indicative for B-cells switching under IFN-γ/Th1 influence. These results suggest that alongside the induction of regulatory T cell responses, also immune deviation toward Th1 may account for therapeutic efficacy. As we could not detect increased levels of IL-10 or TGF-β1 after TCIT, induction of Tr1/Th3 cells seems unlikely.

Subcutaneous injections induced a different type of response, including a clear induction of IL-10, which was accompanied by IL-4, IL-5, and IL-13 secretion. An explanation for this cytokine pattern may be a beginning self-regulation of Th2 inflammation at this time point, rather than a fully formed Tr1 response 45.

Both TCIT and SCIT induced an increase in FOXP3+ CD4+ Treg cells, indicating the involvement of regulatory T cells in the therapeutically active mechanism (Fig. 7). In addition to (i) induction of regulatory T cells and/or (ii) immune deviation to a Th1-biased response type, (iii) blocking antibodies are suggested as an immunological mechanism underlying the effects of SIT 46. However, measuring the antibody titers in our mouse model revealed only a modest increase in IgG1 for SCIT and in IgG2a for SCIT and TCIT, making blocking antibodies as major mechanism unlikely.

Interestingly, our data also point to a substantial difference between the underlying immunological mechanisms of TCIT and SCIT. Both approaches were equally effective with respect to their therapeutic efficacy; however, the immune profile of TCIT proved to be superior concerning the induction of systemic inflammatory side effects, leading to decreased Th1 and Th17 cytokine levels. Moreover, TCIT did not trigger an exacerbation of the allergic response, such as the striking increase of Th2 cytokines after SCIT. It is conceivable that the differences in outcome parameters between TCIT and SCIT may reflect the lower dose of allergen actually delivered by TCIT. *In vitro* experiments with dermatomed porcine skin samples revealed that only 8.2% of the applied dose of FITC-BSA was delivered within 24 h. This is in agreement with data from our mouse studies showing that after s.c. injection, up to 80% of dendritic cells in draining lymph nodes actually carry the antigen compared with 10-20% (depending on pulse number) following TCI. However, we could also demonstrate that different subsets of DCs are targeted 36, suggesting that the observed differences between TCIT and SCIT may not only reflect a dose effect.

In summary, we demonstrated for the first time the applicability of laser microporation for TCIT. Due to the efficient and rapid antigen uptake via small areas of porated skin, this painless method has the potential for a transcutaneous-specific immunotherapy approach with both a high safety profile and an optimal complicance.

## Authors' contributions

DB, MH, and SK performed experiments and data acquisition. EW oversaw the conduct of the study and participated in data interpretation. WK and CHK performed histology. RW and SS designed the study, performed data analysis and interpretation, and drafted the manuscript. CB gave technical assistance and took part in study conception. JT performed study conception, data interpretation, and manuscript drafting. All authors contributed to revising the manuscript and approved the final version.

## Conflict of interest

DB, MH, SK, EEW, and SS have received funding from the Austrian Science Fund. RW and JT have received research support from the Christian Doppler Research Association and from Biomay AG, Vienna, Austria. CB is CEO of Pantec Biosolutions AG. WDK and CHK declare no conflict of interest.

## Supporting Information

Figure S1 Representative HE stained paraffin sections of lungs from mice either treated subcutaneously (SCIT), transcutaneously (TCIT), or which remained untreated (control).

## References

[b1] Asher MI, Montefort S, Bjorksten B, Lai CK, Strachan DP, Weiland SK (2006). Worldwide time trends in the prevalence of symptoms of asthma, allergic rhinoconjunctivitis, and eczema in childhood: ISAAC phases one and three repeat multicountry cross-sectional surveys. Lancet.

[b2] Calderon M, Cardona V, Demoly P (2012). One hundred years of allergen immunotherapy European academy of allergy and clinical immunology celebration: review of unanswered questions. Allergy.

[b3] Freeman J (1911). Further observations on the treatment of hay fever by hypodermic inoculations of pollen vaccine. Lancet.

[b4] Noon L (1911). Prophylactic inoculation against hay fever. Lancet.

[b5] Eifan AO, Shamji MH, Durham SR (2011). Long-term clinical and immunological effects of allergen immunotherapy. Curr Opin Allergy Clin Immunol.

[b6] Jacobsen L, Niggemann B, Dreborg S, Ferdousi HA, Halken S, Host A (2007). Specific immunotherapy has long-term preventive effect of seasonal and perennial asthma: 10-year follow-up on the PAT study. Allergy.

[b7] Cox L, Calderon MA (2010). Subcutaneous specific immunotherapy for seasonal allergic rhinitis: a review of treatment practices in the US and Europe. Curr Med Res Opin.

[b8] Werfel T (2009). Epicutaneous allergen administration: a novel approach for allergen-specific immunotherapy?. J Allergy Clin Immunol.

[b9] More DR, Hagan LL (2002). Factors affecting compliance with allergen immunotherapy at a military medical center. Ann Allergy Asthma Immunol.

[b10] Frew AJ (2010). Allergen immunotherapy. J Allergy Clin Immunol.

[b11] Canonica GW, Bousquet J, Casale T, Lockey RF, Baena-Cagnani CE, Pawankar R (2009). Sub-lingual immunotherapy: World Allergy Organization position paper 2009. Allergy.

[b12] Frew AJ (2008). Sublingual immunotherapy. N Engl J Med.

[b13] Senna G, Lombardi C, Canonica GW, Passalacqua G (2010). How adherent to sublingual immunotherapy prescriptions are patients? The manufacturers' viewpoint. J Allergy Clin Immunol.

[b14] Razafindratsita A, Saint-Lu N, Mascarell L, Berjont N, Bardon T, Betbeder D (2007). Improvement of sublingual immunotherapy efficacy with a mucoadhesive allergen formulation. J Allergy Clin Immunol.

[b15] Streilein JW (1983). Skin-associated lymphoid tissues (SALT): origins and functions. J Invest Dermatol.

[b16] Gutowska-Owsiak D, Ogg GS (2012). The epidermis as an adjuvant. J Invest Dermatol.

[b17] Kupper TS, Fuhlbrigge RC (2004). Immune surveillance in the skin: mechanisms and clinical consequences. Nat Rev Immunol.

[b18] Steinman RM, Banchereau J (2007). Taking dendritic cells into medicine. Nature.

[b19] Blamoutier P, Blamoutier J, Guibert L (1959). [Treatment of pollinosis with pollen extracts by the method of cutaneous quadrille ruling]. Presse Med.

[b20] Pautrizel R, Cabanieu G, Bricaud H, Broustet P (1957). [Allergenic group specificity & therapeutic consequences in asthma; specific desensitization method by epicutaneous route]. Sem Hop.

[b21] Agostinis F, Forti S, Di Berardino F (2010). Grass transcutaneous immunotherapy in children with seasonal rhinoconjunctivitis. Allergy.

[b22] Dupont C, Kalach N, Soulaines P, Legoue-Morillon S, Piloquet H, Benhamou PH (2010). Cow's milk epicutaneous immunotherapy in children: a pilot trial of safety, acceptability, and impact on allergic reactivity. J Allergy Clin Immunol.

[b23] Mondoulet L, Dioszeghy V, Ligouis M, Dhelft V, Dupont C, Benhamou PH (2010). Epicutaneous immunotherapy on intact skin using a new delivery system in a murine model of allergy. Clin Exp Allergy.

[b24] Senti G, Graf N, Haug S, Ruedi N, von Moos S, Sonderegger T (2009). Epicutaneous allergen administration as a novel method of allergen-specific immunotherapy. J Allergy Clin Immunol.

[b25] Senti G, von Moos S, Tay F, Graf N, Sonderegger T, Johansen P (2012). Epicutaneous allergen-specific immunotherapy ameliorates grass pollen-induced rhinoconjunctivitis: a double-blind, placebo-controlled dose escalation study. J Allergy Clin Immunol.

[b26] Von Moos S, Johansen P, Waeckerle-Men Y, Mohanan D, Senti G, Haffner A (2012). The contact sensitizer diphenylcyclopropenone has adjuvant properties in mice and potential application in epicutaneous immunotherapy. Allergy.

[b27] Bachhav YG, Summer S, Heinrich A, Bragagna T, Bohler C, Kalia YN (2010). Effect of controlled laser microporation on drug transport kinetics into and across the skin. J Control Release.

[b28] Yu J, Bachhav YG, Summer S, Heinrich A, Bragagna T, Bohler C (2010). Using controlled laser-microporation to increase transdermal delivery of prednisone. J Control Release.

[b29] Yu J, Kalaria DR, Kalia YN (2011). Erbium:YAG fractional laser ablation for the percutaneous delivery of intact functional therapeutic antibodies. J Control Release.

[b30] Karnovsiky MJ (1965). A formaldehyde-glutaraldehyde fixative of high osmolarity for use in electron microscopy. J Cell Biol.

[b31] Stoecklinger A, Grieshuber I, Scheiblhofer S, Weiss R, Ritter U, Kissenpfennig A (2007). Epidermal langerhans cells are dispensable for humoral and cell-mediated immunity elicited by gene gun immunization. J Immunol.

[b32] Hartl A, Weiss R, Hochreiter R, Scheiblhofer S, Thalhamer J (2004). DNA vaccines for allergy treatment. Methods.

[b33] Stoecklinger A, Scheiblhofer S, Roesler E, Lang A, Fastner G, Sedlmayer F (2012). T cell epitopes of the timothy grass pollen allergen Phl p 5 of mice and men and the detection of allergen-specific T cells using class II ultimers. Int Arch Allergy Immunol.

[b34] Roesler E, Weiss R, Weinberger EE, Fruehwirth A, Stoecklinger A, Mostbock S (2009). Immunize and disappear-safety-optimized mRNA vaccination with a panel of 29 allergens. J Allergy Clin Immunol.

[b35] Gabler M, Scheiblhofer S, Kern K, Leitner WW, Stoecklinger A, Hauser-Kronberger C (2006). Immunization with a low-dose replicon DNA vaccine encoding Phl p 5 effectively prevents allergic sensitization. J Allergy Clin Immunol.

[b36] Weiss R, Hessenberger M, Kitzmueller S, Bach D, Weinberger EE, Krautgartner WD (2012). Transcutaneous vaccination via laser microporation. J Control Release.

[b37] Taube C, Duez C, Cui ZH, Takeda K, Rha YH, Park JW (2002). The role of IL-13 in established allergic airway disease. J Immunol.

[b38] Bal SM, Ding Z, van Riet E, Jiskoot W, Bouwstra JA (2010). Advances in transcutaneous vaccine delivery: do all ways lead to Rome?. J Control Release.

[b39] Senti G, Johansen P, Kundig TM (2009). Intralymphatic immunotherapy. Curr Opin Allergy Clin Immunol.

[b40] Farage MA, Miller KW, Elsner P, Maibach HI (2008). Functional and physiological characteristics of the aging skin. Aging Clin Exp Res.

[b41] Wesley NO, Maibach HI (2003). Racial (ethnic) differences in skin properties: the objective data. Am J Clin Dermatol.

[b42] Bachhav YG, Heinrich A, Kalia YN (2011). Using laser microporation to improve transdermal delivery of diclofenac: increasing bioavailability and the range of therapeutic applications. Eur J Pharm Biopharm.

[b43] Zech NH, Murtinger M, Uher P (2011). Pregnancy after ovarian superovulation by transdermal delivery of follicle-stimulating hormone. Fertil Steril.

[b44] Senti G, von Moos S, Kundig TM (2011). Epicutaneous allergen administration: is this the future of allergen-specific immunotherapy?. Allergy.

[b45] Jankovic D, Kugler DG, Sher A (2010). IL-10 production by CD4+ effector T cells: a mechanism for self-regulation. Mucosal Immunol.

[b46] Strait RT, Morris SC, Finkelman FD (2006). IgG-blocking antibodies inhibit IgE-mediated anaphylaxis in vivo through both antigen interception and Fc gamma RIIb cross-linking. J Clin Invest.

