# Supplementary material for: Transcutaneous immunotherapy via laser-generated micropores efficiently alleviates allergic asthma in Phl p 5–sensitized mice
Source: Allergy. 2012 Sep 5;67(11):1365–74. doi: 10.1111/all.12005 (PMC3532610; doi:10.1111/all.12005)
Supplement: Figure S1 — Representative HE stained paraffin sections of lungs from mice either treated subcutaneously (SCIT), transcutaneously (TCIT), or which remained untreated (control). [file all0067-1365-SD1.docx]

# Transcutaneous immunotherapy via laser-generated micropores efficiently alleviates allergic asthma in Phl p 5 sensitized mice

D. Bach^1*^, R. Weiss^1*^, M. Hessenberger^1^, S. Kitzmueller^1^, E. E. Weinberger^1^, W. D. Krautgartner^2^, C. Hauser-Kronberger^3^, C. Boehler^4^, J. Thalhamer^1^ and S. Scheiblhofer^1^

^1^Department of Molecular Biology, Division of Allergy and Immunology, University of Salzburg, Austria

^2^Department of Organismic Biology, Division of Light & Electron Microscopy, University of Salzburg, Austria

^3^Department of Pathology, University Hospital Salzburg, Paracelsus Medical University, Salzburg, Austria

^4^Pantec Biosolutions AG, Ruggell, Liechtenstein

*These authors contributed equally.

Corresponding author: Josef Thalhamer, PhD, University of Salzburg, Department of Molecular Biology, Hellbrunnerstrasse 34, 5020 Salzburg, Austria. Tel.: +43 662 8044 5737; Fax: +43 662 8044 5751; E-mail: [Josef.Thalhamer@sbg.ac.at](mailto:Josef.Thalhamer@sbg.ac.at)

## Methods

2µm paraffin sections of 4% formaldehyde fixed lungs were prepared and stained with haematoxylin/eosin using standard methods. Slides were scored by an investigator who was blind to the treatment groups. Bronchioles and vessels were counted as “normal” or “abnormal”, depending on the amount of surrounding inflammatory infiltrate as previously described ([1](#_ENREF_1)) and the number of abnormal peribronchial/perivascular spaces was divided by the total number of peribronchial/perivascular spaces to generate a lung pathology score.

Statistical significance between groups was assessed by Kruskal-Wallis test followed by Dunn’s Multiple Comparison Test (alpha = .05) using GraphPad Prism 5.

## Results

Both treatment groups showed a tendency towards reduced levels of peribronchial and perivascular mononuclear infiltrate. SCIT showed marginally better results than TCIT, but no statistical significance was reached. Supplementary Figure 1A shows representative examples from the different treatment groups, and lung pathology scores of individual mice are shown in supplementary Fig. 1B.


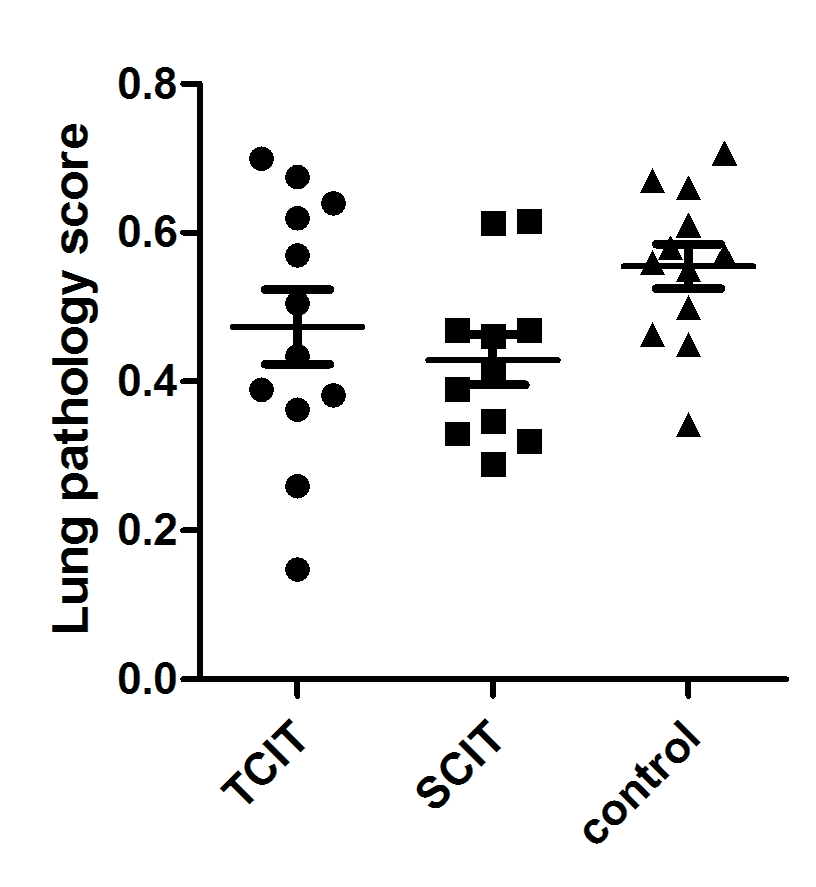

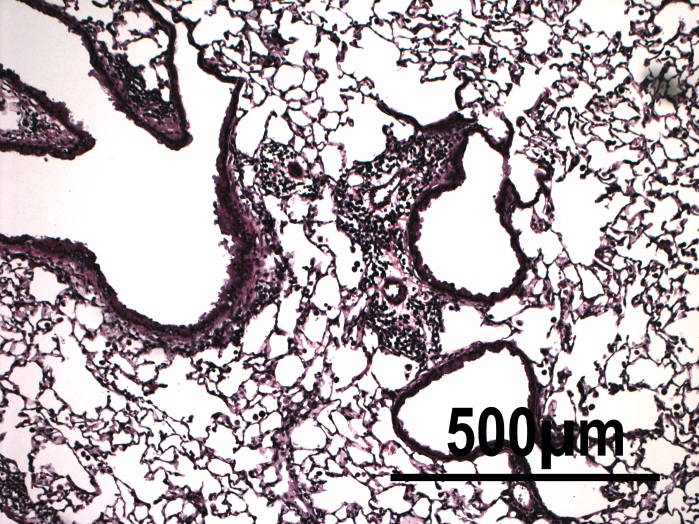

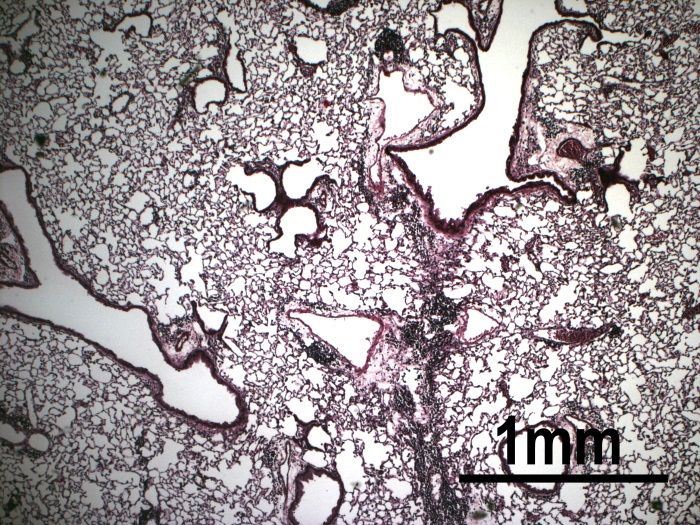

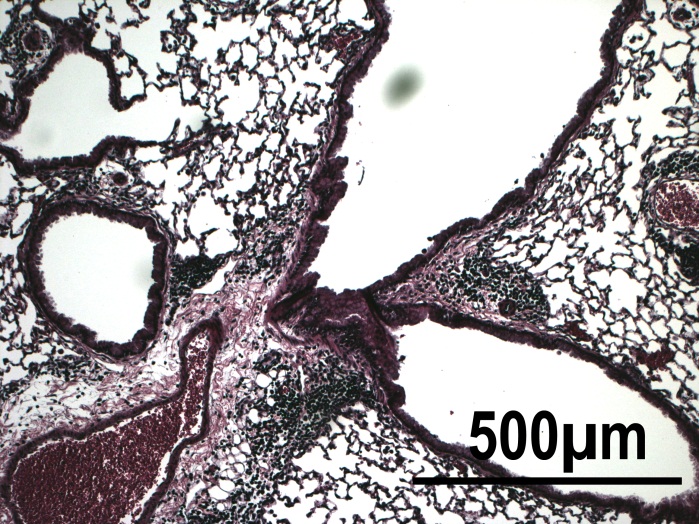

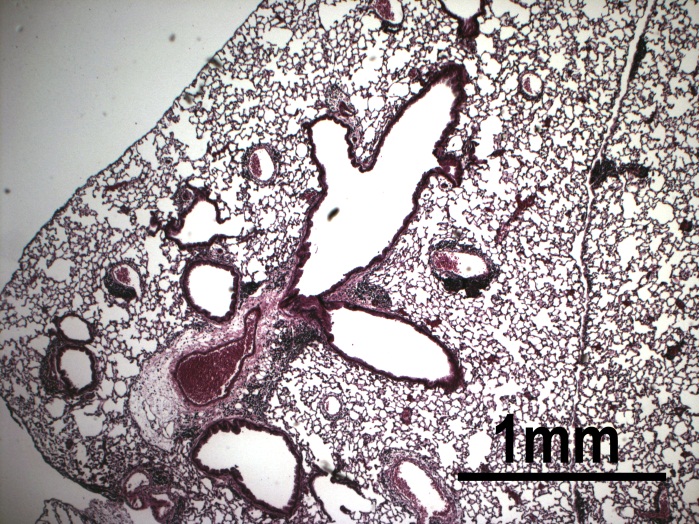

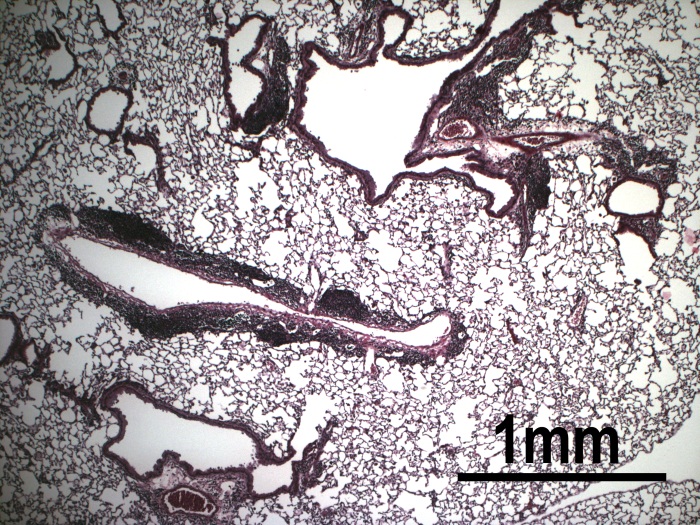

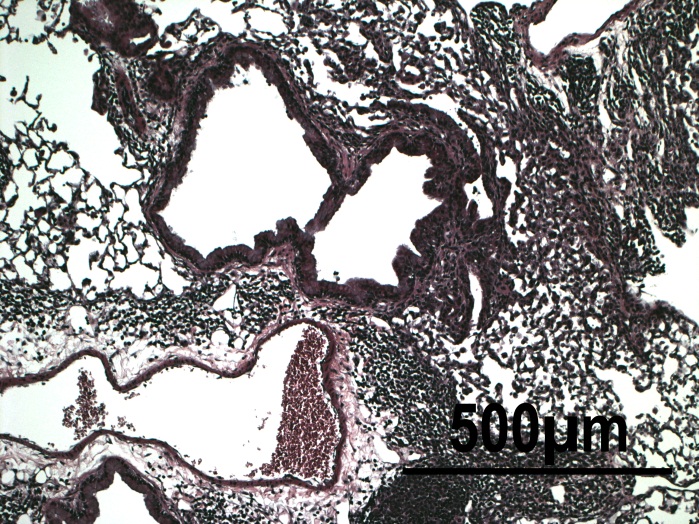


Supplementary Fig. 1. A) Representative HE stained paraffin sections of lungs from mice either treated subcutaneously (SCIT), transcutaneously (TCIT), or which remained untreated (control). Left panels: original magnification 40x, right panels: original magnification 100x.

B) Lung pathology scores of individual mice (n=12).

B

A

TCIT

SCIT

control

## References

1. Haeberle HA, Nesti F, Dieterich HJ, Gatalica Z, Garofalo RP. Perflubron reduces lung inflammation in respiratory syncytial virus infection by inhibiting chemokine expression and nuclear factor-kappa B activation. *Am J Respir Crit Care Med* 2002;**165**(10):1433-1438.
